# Are renal tumors associated with lithium treatment?

**DOI:** 10.1186/2194-7511-2-6

**Published:** 2014-04-16

**Authors:** Ross J Baldessarini, Leonardo Tondo

**Affiliations:** International Consortium for Bipolar Disorder Research, Mailman Research Center–McLean Hospital, Harvard Medical School, Boston, MA 02115 USA

## Editorial

Zaidan et al. (2014) recently identified solid renal tumors by sonographic scanning among 170 patients in Parisian renal disorder programs who had been treated with lithium for an average of 21.4 years and developed decreased renal function. In such patients, the observed rate of renal tumors was high, at 14/170 (8.24%), and half of the tumors were considered malignant (4.12%). The observed rate of solid renal tumors was nearly six times greater than among patients with chronic renal disease (matched for age, sex, and glomerular filtration rate) but not known to have been exposed to lithium (8.24% vs. 5/340 or 1.47%), and the risk among all observed patients with renal dysfunction (19/510 or 2.73%) was over three times less than with lithium treatment (2.73% vs. 8.24%). These rates were reported to be far greater than in the general population. However, risks among lithium-treated patients without renal impairment were not reported. The report proposes that there is an increased risk of renal tumors in association with long-term exposure to lithium salts among patients with impaired renal function.

These striking observations, if validated, are of considerable concern, particularly as long-term treatment with lithium has been associated with risk of pathological and pathophysiological changes in renal function that call for regular, routine clinical monitoring of indices of renal function among patients treated long-term with lithium (Bauer et al. 2006; Baldessarini 2013). Rates of severe impairment of renal function among lithium-treated patients may be as low as 0.5% (McKnight et al. 2012). Given an estimated risk of solid renal tumors of 8.24% (and of malignancies of about 4.12%) of lithium-treated patients with significant renal impairment (Zaidan et al. 2014), a tentative estimate of risk of kidney tumors among all patients exposed to lithium long-term would be approximately 0.041% (0.0824 × 0.005), and of cancers of about 0.020%. This estimate suggests a risk of one case of solid renal tumor in approximately 2,400 lithium-treated patients or of a malignancy in approximately 4,800 lithium-treated patients. These rates indicate that large samples will be required to evaluate risks among lithium-treated patients. These and other medical risks are to be balanced against the extraordinary body of evidence that lithium, more than any other mood stabilizer, not only has short-term antimanic effects but can also reduce risks of recurrences of various components of bipolar disorder and may reduce the risks of suicide as well as of mortality associated with cardiovascular and respiratory illnesses, which are more frequent in bipolar patients compared to the general population (Bauer et al. 2006; Ahrens et al. 1995; Baldessarini et al. 2006; Grof and Müller-Oerlinghausen 2009).

The preceding considerations suggest that the findings reported by Zaidan et al. (2014) require further study, ideally in large samples of patients exposed to lithium treatment, stratified for renal functional status, lithium exposure, and perhaps age. Clinically, the findings underscore the importance of close medical monitoring of patients treated with lithium, especially for more than several years, and consideration of renal scanning examinations for cysts and tumors among those with clinically identified, progressive impairment of renal function. Our impression, based on consulting international colleagues who lead large mood disorder clinical programs, is that no reports of renal cancers have emerged in a collective experience involving thousands of psychiatric patients treated long-term with lithium (IGSLI centers, http://www.igsli.org; personal written communication, 28 March 2014; and clinical records of the Lucio Bini Mood Disorders Centers, Rome and Cagliari, March 2014). Nevertheless, in view of the potential seriousness of the new findings, it would be of interest to organize an international, register-based, cohort study to investigate the hypothesis that the risk of renal tumors is increased among patients exposed to lithium, including in subgroups defined by lithium exposure and renal functional status.
